# Supplementary material for: Computational analysis of therapeutic potential for simplified Piper spp.- derived medicinal mixtures in anxiety, sleep, pain and seizure
Source: Front Pharmacol. 2026 Aug 3;17:1750146. doi: 10.3389/fphar.2026.1750146 (PMC13478742; doi:10.3389/fphar.2026.1750146)
Supplement: Supplementary file 1 [file Supplementaryfile1.pdf]

Supplemental Figure A.

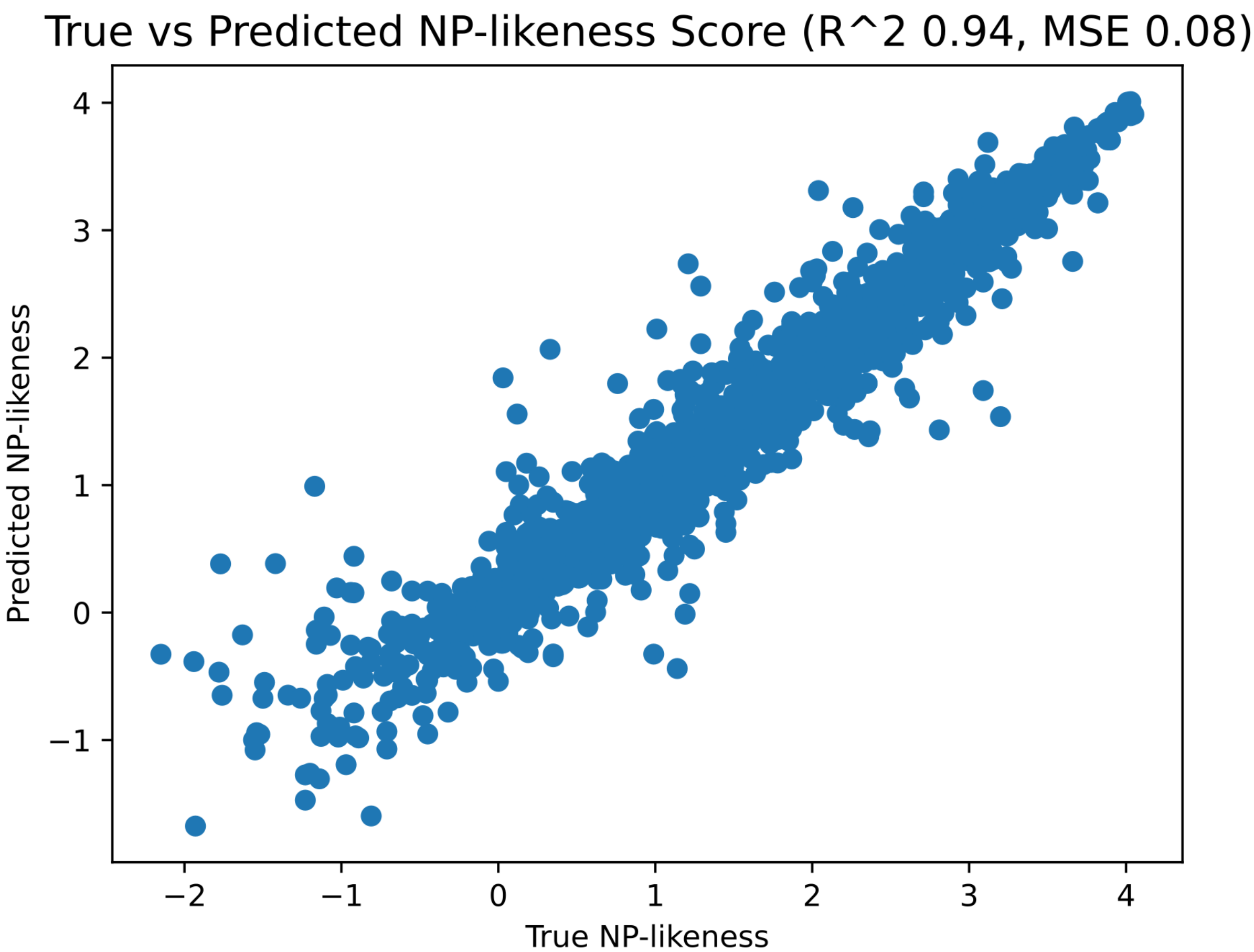

Supplemental Figure B.

Actual	Truncated	**Figure**
17-methoxy-5,7-dioxa-1-azoniapentacyclo[11.8.0.03,11.04,8.014,19]henicosa-1(13),2,4(8),9,11,14,16,18-octaen-16-ol	17-methoxy-protoberberine*	Figure 4A
3alpha,4alpha-epoxy-5beta-pipermethystine	3α,4α-epoxy-5β-pipermethystine	Figure 4A
(1r,13s,21r)-16,17-dimethoxy-13-oxido-5,7-dioxa-13-azoniapentacyclo[11.8.0.02,10.04,8.015,20]henicosa-2,4(8),9,15(20),16,18-hexaen-21-ol	dimethoxy-oxido-protoberberine*	Figure 4A
(23s)-23-ethoxy-24-methyl-5,7,18,20-tetraoxa-24-azahexacyclo[11.11.0.02,10.04,8.014,22.017,21]tetracosa-1(13),2,4(8),9,11,14(22),15,17(21)-octaene	ethoxy-methyl-protoberberine derivative*	Figure 4A
1-(4-hydroxy-3,4-dihydro-2h-pyridin-1-yl)-3-phenylpropan-1-one	hydroxypyridinyl phenylpropanone 1*	Figure 4A
1-(4-hydroxypiperidin-1-yl)-3-phenylpropan-1-one	hydroxypyridinyl phenylpropanone 2*	Figure 4A
1-[(4r)-4-hydroxy-3,4-dihydro-2h-pyridin-1-yl]-3-phenylpropan-1-one	hydroxypyridinyl phenylpropanone 3*	Figure 4A
[(3s)-6-oxo-1-(3-phenylpropanoyl)-2,3-dihydropyridin-3-yl] acetate	phenylpropanoyl-dihydropyridinyl acetate*	Figure 4A
[(1s,5r,6s)-2-oxo-3-(3-phenylpropanoyl)-7-oxa-3-azabicyclo[4.1.0]heptan-5-yl] acetate	phenylpropanoyl-oxazabicyclo acetate*	Figure 4A
4,5,17-trimethoxy-11-methyl-2-oxa-11-azatetracyclo[8.7.1.03,8.014,18]octadeca-1(17),3(8),4,6,9,14(18),15-heptaene-12,13-dione	trimethoxy-oxa-azatetracyclo dione*	Figure 4A
(11bs)-2,3,9-trimethoxy-7,11b,12,13-tetrahydro-6h-isoquinolino[2,1-a]quinolin-10-ol	trimethoxyisoquinolinoquinolinol*	Figure 4A
(1,7,7-trimethyl-2-bicyclo[2.2.1]heptanyl) 3-(2-methoxyphenyl)prop-2-enoate	trimethyl-bicyclo-heptanyl-benzodioxol-methoxyphenyl-propenoate	Figure 4A
(1,7,7-trimethyl-2-bicyclo[2.2.1]heptanyl) 3-(1,3-benzodioxol-5-yl)prop-2-enoate	trimethyl-bicyclo-heptanyl-benzodioxol-propenoate 1*	Figure 4A
[(1r,2s,4r)-1,7,7-trimethyl-2-bicyclo[2.2.1]heptanyl] (e)-3-(1,3-benzodioxol-5-yl)prop-2-enoate	trimethyl-bicyclo-heptanyl-benzodioxol-propenoate 2*	Figure 4A
(s)-2,3-dihydro-5-hydroxy-7-methoxy-2-phenyl-4-benzopyrone	dihydro-hydroxy-methoxy-phenyl-benzopyrone*	Figure 4A
2-[2-(1,3-benzodioxol-5-yl)ethenyl]-4-methoxy-2,3-dihydropyran-6-one	benzodioxolyl-ethenyl-methoxy-dihydropyranone*	Figure 4A
2-[(e)-2-(3,4-dimethoxyphenyl)ethenyl]-4-methoxy-2,3-dihydropyran-6-one	dimethoxyphenyl-ethenyl-methoxy-dihydropyranone*	Figure 4A
(2s)-4-methoxy-2-[2-(4-methoxyphenyl)ethyl]-2,3-dihydropyran-6-one	methoxy-methoxyphenyl-ethyl-dihydropyranone*	Figure 4A
4-methoxy-6-[(2s,3r)-3-(4-methoxyphenyl)oxiran-2-yl]pyran-2-one	methoxy-methoxyphenyl-oxiranyl-pyranone*	Figure 4A
4-methoxy-2-(2-phenylethenyl)-2,3-dihydropyran-6-one	methoxy-phenylethenyl-dihydropyranone*	Figure 4A
2,2'-dihydroxy-4',6'-dimethoxychalcone	dihydroxy-dimethoxychalcone*	Figure 4A
(e)-3-(2,4-dihydroxyphenyl)-1-(2-hydroxy-4,6-dimethoxyphenyl)prop-2-en-1-one	dihydroxyphenyl-dimethoxyphenyl-propenone*	Figure 4A
(e)-1-(2-hydroxy-4,6-dimethoxyphenyl)-3-(3-hydroxyphenyl)prop-2-en-1-one	dimethoxyphenyl-hydroxyphenyl-propenone*	Figure 4A
(e)-1-(2-hydroxy-4,6-dimethoxyphenyl)-3-(3-methoxyphenyl)prop-2-en-1-one	dimethoxyphenyl-methoxyphenyl-propenone*	Figure 4A
(e)-1-(2-hydroxy-4,6-dimethoxyphenyl)-3-(2-methylphenyl)prop-2-en-1-one	dimethoxyphenyl-methylphenyl-propenone*	Figure 4A
(e)-1-(2-hydroxy-4,6-dimethoxyphenyl)-3-(2-propan-2-ylphenyl)prop-2-en-1-one	dimethoxyphenyl-propanylphenyl-propenone*	Figure 4A
2',4'-dimethylchalconaringenin	dimethylchalconaringenin*	Figure 4A
(e)-3-(2,6-dimethylphenyl)-1-(2-hydroxy-4,6-dimethoxyphenyl)prop-2-en-1-one	dimethylphenyl-dimethoxyphenyl-propenone*	Figure 4A
(e)-3-(2-ethylphenyl)-1-(2-hydroxy-4,6-dimethoxyphenyl)prop-2-en-1-one	ethylphenyl-dimethoxyphenyl-propenone*	Figure 4A
6'-hydroxy-2',4'-dimethoxychalcone	hydroxy-dimethoxychalcone*	Figure 4A
2'-hydroxy-2,4',6'-trimethoxychalcone	hydroxy-trimethoxychalcone*	Figure 4A
2',3,4-trihydroxy-4',6'-dimethoxychalcone	trihydroxy-dimethoxychalcone*	Figure 4A

Supplemental Figure B.

Actual	Truncated	**Figure**
[1-(3,4-dimethoxyphenyl)-2-(2,6-dimethoxy-3-prop-2-enylphenoxy)propyl] acetate	dimethoxy-propenylphenoxy-propyl acetate*	Figure 7
(2beta,4beta,5alpha,5abeta)-4-(1,3-benzodioxol-5-yl)-2,3,4,5-tetrahydro-7-methoxy-5-methyl-8h-2,5a-methano-1-benzoxepin-8-one	benzodioxolyl-hydro-methoxy-benzoxepinone*	Figure 7
(2e,4e,11e)-12-(benzo[1,3]dioxol-5-yl)-n-(3-methylbutyl)dodeca-2,4,11-trien-amide	benzodioxolyl-methylbutyl-dodeca-trien-amide*	Figure 7
(2e,4e,6e)-7-(1,3-benzodioxol-5-yl)-1-pyrrolidin-1-ylhepta-2,4,6-trien-1-one	benzodioxolyl-pyrrolidinylhepta-trienone*	Figure 7
(2e,6e)-7-(1,3-benzodioxol-5-yl)-1-(1-pyrrolidinyl)-2,6-heptadien-1-one	benzodioxolyl-pyrrolidinyl-heptadienone*	Figure 7
1-[(2e,4e,8e)-9-(3,4-methylenedioxyphenyl)-2,4,8-nonatrienoyl]pyrrolidine	methylenedioxyphenyl-nonatrienoyl-pyrrolidine*	Figure 7
1-[(2z,4e)-5-(1,3-benzodioxole-5-yl)-1-oxo-2,4-pentadienyl]pyrrolidine	benzodioxoleyl-oxo-pentadienyl-pyrrolidine*	Figure 7
1-[(2e,4s,5r)-4,5-dihydroxy-1-oxo-2-decenyl]piperidine	dihydroxy-oxo-decenyl-piperidine*	Figure 7
1-(3-phenylpropanoyl)-5,6-dihydropyridin-2(1h)-one	phenylpropanoyl-dihydropyridinone*	Figure 7
(e,7s,11r)-3,7,11,15-tetramethylhexadec-2-en-1-ol	tetramethylhexadecenol*	Figure 7
2-(4-ethenyl-4-methyl-3-prop-1-en-2-ylcyclohexyl)propan-2-ol	ethenyl-methyl-propenylcyclohexyl-propanol*	Figure 7
azulene, 1,2,3,4,5,6,7,8-octahydro-1,4-dimethyl-7-(1-methylethenyl)-, (1s,4s,7r)-	azulene*	Figure 7
bicyclo(3.1.1)hept-2-ene-2-carboxaldehyde, 6,6-dimethyl-, (1s)-	dimethyl-bicyclo-heptene-carboxaldehyde*	Figure 7
[(1r,2s)-2-(3,4-dimethoxy-6-oxo-1-prop-2-enylcyclohexa-2,4-dien-1-yl)-1-(3,4-dimethoxyphenyl)propyl] acetate	cyclohexadienyl-dimethoxyphenylpropyl acetate*	Figure 7
1-[(2e,4e,8e)-9-(3,4-methylenedioxyphenyl)-2,4,8-nonatrienoyl]pyrrolidine	*pyrrolidine 1	Figure 8A
1-[(2e,8e)-9-(3,4-methylenedioxyphenyl)-2,8-nonadienoyl]pyrrolidine	*pyrrolidine 2	Figure 8A
(s)-2,3-dihydro-5-hydroxy-7-methoxy-2-phenyl-4-benzopyrone (10)	*benzopyrone	Figure 8B
2'-hydroxy-4,4',6'-trimethoxychalcone	*trimethoxychalcone	Figure 8B
(7s,8r)-4-hydroxy-4',7-epoxy-8,3'-neolignan-(7e)-ene	*neolignan-3	Figure 8B
(7s,8r)-4-hydroxy-8',9'-dinor-4',7-epoxy-8,3'-neolignan-7'-aldehyde	*neolignan-2	Figure 8B
1-[1-oxo-3(3,4-methylenedioxy-5-methoxyphenyl)-2zpropenyl] piperidine	*piperidine-2	Figure 8B
(7r,8r)-3,4-methylenedioxy-4',7-epoxy-8,3'-neolignan-7'e-ene	*neolignan-4	Figure 8B
methyl-(7r,8r)-4-hydroxy-8',9'-dinor-4',7-epoxy-8,3'-neolignan-7'-ate	*neolignan-1	Figure 8B
1-(1-oxo-3-phenyl-2e-propenyl)piperidine	*piperidine-3	Figure 8B
(e,7s,11r)-3,7,11,15-tetramethylhexadec-2-en-1-ol	*tetramethylhexadec	Figure 8B
azulene, 1,2,3,4,5,6,7,8-octahydro-1,4-dimethyl-7-(1-methylethenyl)-, (1s,4s,7r)-	*azulene	Figure 8C
bicyclo(3.1.1)hept-2-ene-2-carboxaldehyde, 6,6-dimethyl-, (1s)-	*bicyclo-carboxaldehyde	Figure 8C
2-(4-ethenyl-4-methyl-3-prop-1-en-2-ylcyclohexyl)propan-2-ol	*propan-2-ol	Figure 8C
(4r,10s)-4,10-dimethyl-7-propan-2-yltricyclo[4.4.0.01,5]decan-4-ol	*propan-2-yltricyclo*decan-4-ol	Figure 8C

Supplemental Figure C.

Abbreviation	Full Term
ALogP	Atom-based log partition coefficient
ANOVA	Analysis of Variance
BioPhytMol	Bioactive Phytochemicals Molecular Database
ChEMBL	Chemical Database of Bioactive Molecules with Drug-like Properties
CID	Compound Identifier
ECFP4	Extended-Connectivity Fingerprints (type 4)
ETCM	Encyclopedia of Traditional Chinese Medicine
FDA	U.S. Food and Drug Administration
GABA	Gamma-Aminobutyric Acid
GBIF	Global Biodiversity Information Facility
GIMS	Global Integrative Medical Systems
hpf	Hours Post-Fertilization
IMPPAT	Indian Medicinal Plants, Phytochemistry And Therapeutics
InChIKey	International Chemical Identifier (hashed)
ISCA	In Silico Convergence Analysis
KEGG	Kyoto Encyclopedia of Genes and Genomes
KL	Kavalactones
KTKP	Korean Traditional Knowledge Portal
MedPServer	Medicinal Plant Server
MEEF	Minimal Essential Effective Formulations
NANPDB	Northern African Natural Products Database
Noldus	Behavioral tracking software (EthoVision XT)
NP-likeness	Natural Product-likeness
PharmDB-K	Pharmacological Database of Korean Medicinal Plants
PhAROS™	Phytomedicine Analytics for Research Optimization at Scale
PTSD	Post-Traumatic Stress Disorder
PubChem	Public Chemical Molecule Database
QED	Quantitative Estimate of Drug-likeness
RDKit	Open-source cheminformatics software
RO5	Lipinski's Rule of Five
SANCDB	South African Natural Compounds Database
SFE	Supercritical Fluid Extraction
SMILES	Simplified Molecular Input Line Entry System
SPME	Solid Phase Micro Extraction
spp.	Species (plural)
TCMID	Traditional Chinese Medicine Integrated Database
TIPdb	Taiwanese Indigenous Plant Database
TM-MC	Northeast Asian Traditional Medicine Database
